# Mio-Pliocene piracy, relict landscape and drainage reorganization in the Namcha Barwa syntaxis zone of eastern Himalaya

**DOI:** 10.1038/s41598-019-54052-x

**Published:** 2019-11-26

**Authors:** Nilesh Kumar Jaiswara, Prabha Pandey, Anand K. Pandey

**Affiliations:** Academy of Scientific Innovation and Research, CSIR-National Geophysical Research Institute, Uppal Road, Hyderabad, 500007 India

**Keywords:** Natural hazards, Geomorphology, Tectonics

## Abstract

The presence of unique elevated low relief relict landscape in the transient Dibang catchment, at the orographic edge of Tibet-Himalaya in the tectonically active Namcha Barwa syntaxial zone, is modelled to understand evolving regional landscape, drainage reorganization and tectonics. This elevated low relief landscape represents a Mio-Pliocene abandoned paleo-channel of the Yarlung river, which was captured by the headward eroding Siang river owing to >600 m base level advantage. The river capture caused isolation of the Dibang river, which evolved as a transient parched catchment since 3–6 Ma after loss of ~17 times drainage area and 4–17 times discharge. The drainage area and discharge gained by the Siang river triggered enormous incision causing aneurysm leading to the accelerated growth of the Tsangpo gorge and affected regional tectonics. This paleo-drainage reorganization is reflected in the Mio-Pliocene sedimentation pattern in the southern Tibet-Himalaya and foreland basins.

## Introduction

The landscape evolution in an active mountain belt is a function of competing processes of tectonic uplift and erosion into positive feedback by the drainage system^1–8^. The clues of landscape evolution in mountainous terrain are often preserved in elevated low relief landscape of abandoned channel, which develops in response to drainage capture and divide migration, leading to *in-situ* development of low relief upland in a transient system^9–12^. The upstream drainage area loss of abandoned channel would reduce the drainage discharge leading to its *in-situ* evolution as parched low relief landscape with decreased erosion potential of the channel leading to a net surface uplift viz. erosion [E] <rock uplift [U]^9,13,14^. Alternatively, the accelerated incision of preexisting low relief landscape by perturbation in uplift-erosion feedback due to changing tectonic uplift or climate change can also result in the development of elevated low relief landscape^8,14–16^. The gradational disequilibrium in the beheaded/abandoned drainage basin results in transient signals (slope-break knickpoints) with reference to the regional base level in response to the gain in net surface uplift that can be identified in the regional landscape pattern^17–23^. Further, the regional drainage reorganization in tectonically active terrain leads to the regional base level change and large-scale drainage area exchange, impacting the evolution of topographic relief and drainage discharge in surrounding basins^24,25^ and such drainage dynamics needs a careful interpretation of river profiles analysis^8,11–13,26^.

**Figure 1:**
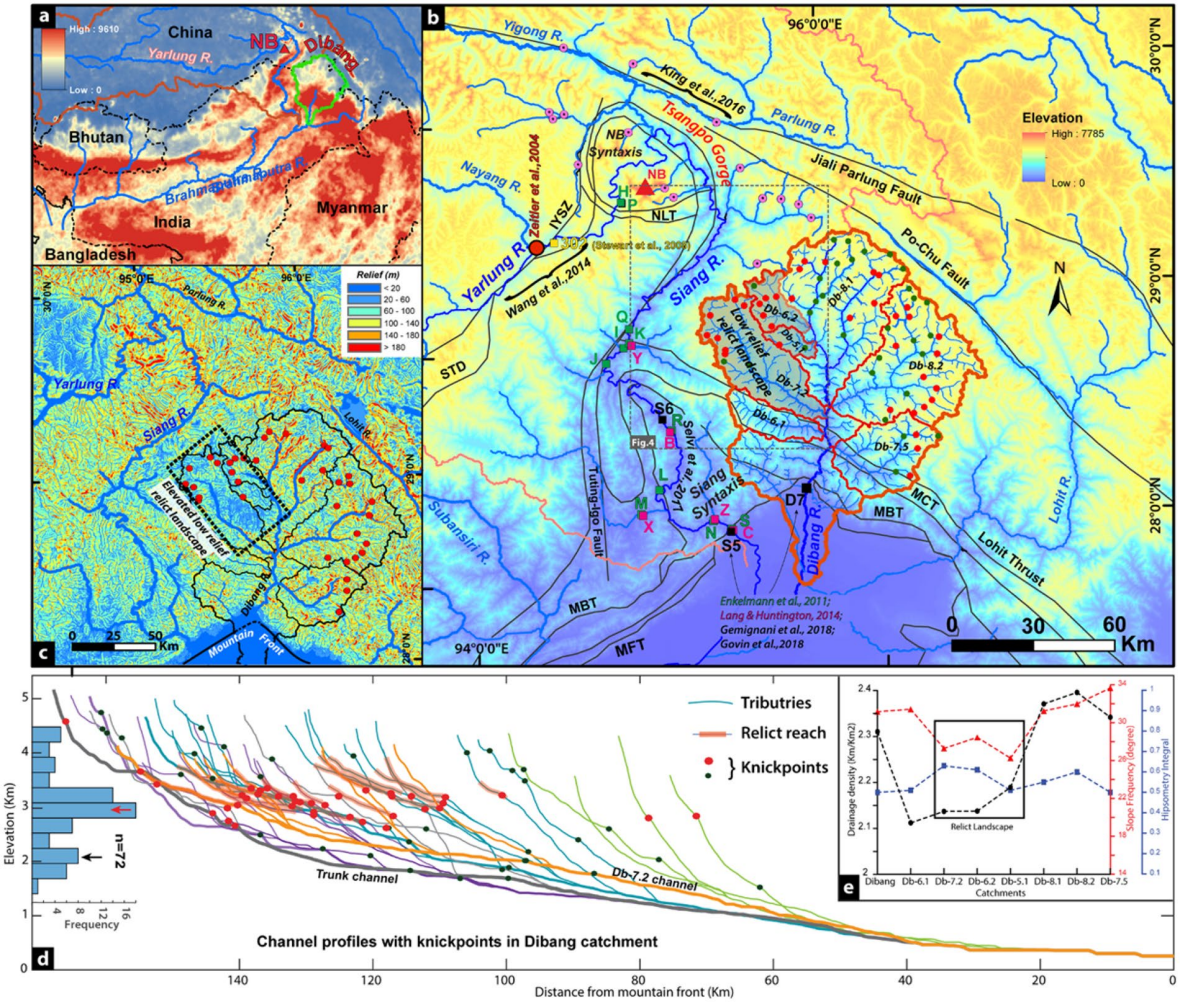
(**a**) The annual TRMM precipitation over the study region in eastern Himalaya and Tibet (http://www.geog.ucsb.edu/~bodo/TRMM/). (**b**) The drainage network in the eastern Himalaya-Tibet with major tectonic boundaries and knick point distribution. (**c**) The low relief landscape in the NW part of Dibang catchment, namely Db-7.2 and Db-6.2 (Also see Fig. SM1) (**d**) Longitudinal channel profiles in Dibang catchment with knickpoints and relict reach. Note the low relief Db-7.2 (orange color) has elevated thalweg. Knickpoint histogram show two prominent distributions at ~3000 m (3k) and ~1900 m. (**e**) The elevated low relief catchment in the Dibang has distinct morphometric characteristics. (IYSZ = Indus Yarlung Suture Zone, NB = Namcha Barwa, NLT = Nam La Thrust, STD = South Tibetan Detachment, MCT = Main Central Thrust, MBT = Main Boundary Thrust and MFT = Main Frontal Thrust).

The Namcha Barwa (NB) syntaxis at the orographic edge of Tibetan plateau and Himalaya has witnessed large-scale drainage reorganizations since Miocene, viz. successive migration of the Yarlung river from the Red river system^1,3^ or the Irrawaddy river prior to the Late Miocene^27^ followed by the drainage reversal and capture of the Yigong-Parlung river by the antecedent Yarlung-Brahmaputra river system during Plio-Pleistocene^28–30^. The presence of relict fluvial landscape at a higher elevation, the provenance study of sediments aided by thermochronology and tectonics have been the important proxies in understanding the large drainage reorganization in the region around the syntaxis^1,3,6,28–35^. The positive feedback effect of active tectonics on erosion and incision vis a vis climate is far over-weighed^5,34,36^ in spite of the region experiencing pronounced precipitation gradient viz. >4000 to 200 mm within a short horizontal distance of ~150 km between the Himalayan front to the Tsangpo gorge at the edge of the Tibet (Fig. 1a).

We observed the presence of a large (>1500 km^2^) anomalous patch of low relief/slope landscape at elevation of >2000 m (constituting the sub-catchments Db 7.2 and 6.2) of Dibang basin adjacent to the Tsangpo gorge across the Yarlung/Siang-Dibang drainage divide (Figs. 1b–d, S1). The low relief sub-catchment (Db 7.2) is elevated by 300–500 m above the Dibang trunk channel and has low slope hanging valleys (cf. Wobus *et al*.^37^) with increasing vertical drop towards downstream segment (Figs. 1d, S1c), which defines the modified preexisting/relict surface^12,13^. This low relief sub-catchment has unique morphometry with lower drainage density, slope frequency, and higher hypsometry integrals in comparison to the rest of the Dibang catchment (Figs. 1e, S1a). This elevated low relief sub-catchment has coincident drainage divide^12^ with tabular top at ~3500 m along the Yarlung/Siang-Dibang divide. The previous regional paleo-drainage reconstruction invariably ignored the isolated and symmetrical (asymmetry index: 51%) Dibang basin^1,29–36,38^. The recent finding of dominant older zircon grains (peak at 10–15 Ma) with high erosion rate and negligible younger (<5 Ma) grains at the Dibang mountain front and conspicuous absence of older zircon (>5 Ma) and dominant younger zircon (<2 Ma age, peak at 2–5 Ma) population in the lower Siang sediments remain poorly explained in terms of provenance^31,35^. To understand the status of the observed elevated low relief zone in the Dibang basin (Figs. 1, S1) and its role in regional drainage reorganization, we modeled the regional landscape to constrain the paleo-base level, surface uplift and timing through transient signals (knickpoint modeling) in the river profile analysis, volume-for time substitution, drainage divide migration, piracy and area loss feedback and regional correlation to comprehend regional landscape evolution. The late Mio-Pliocene drainage capture of the paleo-Yarlung-Dibang river by the Siang river driven the active tectonics and landscape evolution of the syntaxial region of Tibet-eastern Himalaya^4,5^.

## Conceptual Framework

**Figure 2:**
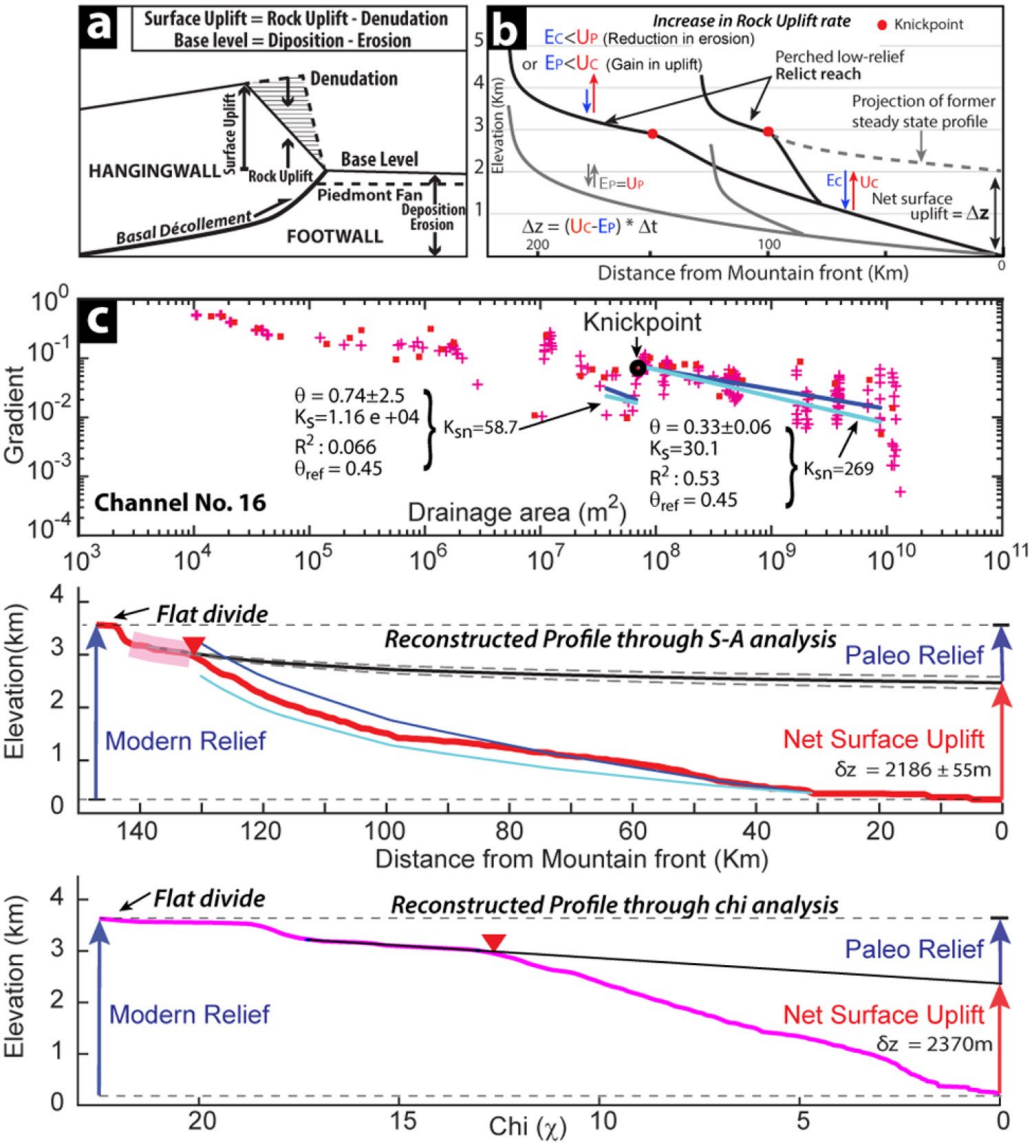
(**a**) The topographic growth in the Himalayan wedge is controlled by the rock uplift and channel denudation in the hanging wall and the footwall elevation defines the base-level of the drainage system. (**b**) Formation and headword migration of slope break knickpoint in tectonically active terrain with reference to pre-perturbation equilibrated profile in response to the variation in uplift-erosion rates during transient state (after Jaiswara *et al*., 2019). (**c**) The reconstruction of paleo-base level of channels in the Dibang catchments using the scaling parameter Ks and mean concavity (θ) of relict reach. The blue and cyan lines show regressed and referenced concavity (θ_ref_), respectively. The reconstructed profile using slope-area analysis with 2σ elevation error and Chi (χ) profile clearly constrains the paleo-base level and the net surface uplift. Note the missing drainage divide peak in the Db-7.2 sub-catchment (Also see Fig. S3).

The landscape in the hanging wall of the Himalayan thrust wedge is controlled by rock uplift and denudation rates, which is dictated by the regional base level in footwall alluvial plain and is consistent since at least late Miocene^38^ (Fig. 2a). Any change in net surface uplift rate in the wedge will lead to the initiation and head-ward propagation of active mobile knickpoints from the mountain front with similar vertical velocity driving the evolution of transient landscape^17–19,22–24,39^. By assuming that mountain front line and drainage area have not changed significantly since the onset of this perturbation, the reconstruction of knickpoint evolution in transient drainage system by projecting the upstream relict profile (relict reach) through the actively adjusting zone can help in estimating net surface uplift by changing surface uplift rate vis a vis steady state^23,26,40,41^ (Fig. 2b). The initiation timing of the change in surface uplift can be constrained using eroded rock volume estimate for given denudation rates^41,42^ and can be tested by knickpoint celerity model^43,44^.

## River Profile Analysis

We performed slope-area analysis on the tributaries of Dibang and Yarlung-Siang river basins and identified ~100 slope-break knickpoints, which separate the longitudinal river profiles into two concave segments (Fig. 1). In the Dibang basin, 72 knickpoints along 43 tributary channels with major slope-break knickpoints at 3005 ± 227 m are identified, we refer these as 3k knickpoints (Fig. 1d). The comparable elevation and radial distribution of these knickpoints suggest a co-genetic origin in the downstream region (Fig. S1a). The slope-area analysis confirms the mobile nature of the knickpoints, which is adjusting as incision wave by headward propagation of local base-level from the mountain front (Fig. S1b). These knickpoints of the Dibang basin were modelled to estimate the net surface uplift and paleo-relief from the reconstructed paleo-base level of the relict reaches (Figs. 1d, S1a). The paleo-base level reconstruction uses the stream power scaling law, which relates local channel slope (*S*) to contributing drainage area (*A*) through the channel parameters of steepness (*k*_*s*_) and concavity (*q*)^44^.

1 $$S={k}_{s}{A}^{-\theta }$$

The steepness indices of 37 selected channels (excluding the DEM artefact) are computed for characterizing the relict reach and actively adjusting landscape downstream of the knickpoints (Table S1). The estimated mean *k*_*sn*_ for the upstream and downstream segments are 114.7 ± 40 m^0.9^ and 294.3 ± 58.6 m^0.9^, respectively. The *k*_*sn*_ distribution clearly suggests the downstream segment of channels exhibit adjustment to the active uplift-erosion regime (Fig. S1a,c). The onset of the new uplift-erosion condition and the paleo-base level can be reconstructed by using the scaling parameters estimated from the relict profile segment above 3k knickpoint. The average concavity index (*θ*_*ref*_) is calculated from the selected upstream relict segments (Table S2; Figs. 2b; S2), and the same is used for calculating *k*_*sn*_ so that effects of geomorphic and hydrologic complications due to varying drainage area are minimized^37,40,45^.

**Figure 3:**
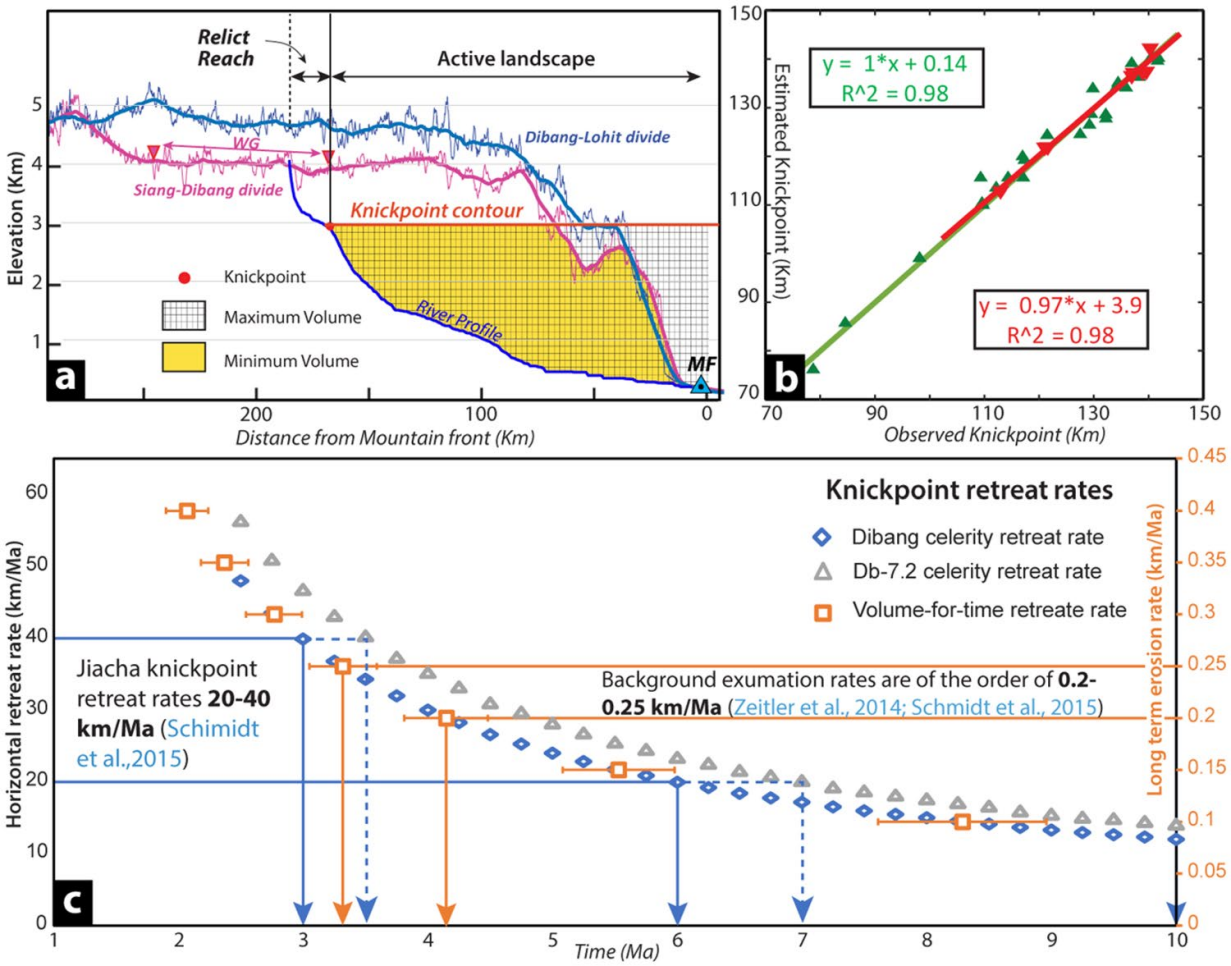
(**a**) The drainage divide profile of Siang-Dibang divide has wind gap and is ~1000 m lower than Dibang-Lohit divide. The 2D-schematic illustration showing maximum and minimum eroded volume derived at 3k knickpoint from channel profile using Norton *et al*., 2008 methodology. (**b**) Correlation of observed and modeled knickpoint distances from mountain front using Celerity model (Crosby & Whipple, 2006; SM1b). The inverted red triangles represent knickpoints in low relief Db-7.2 sub-catchment suggesting their cogenetic origin. (**c**) Comparative horizontal knickpoint retreat rates derived from celerity model and eroded volume methods. Note the elevated low relief region follows the general trend but show faster retreat.

The 22 channel profiles with well-preserved relict reaches are modeled for paleo-channel reconstruction (Table S2). The mean surface uplifts estimated from the reconstructed paleo-base level for slope-area analysis and χ -analysis are 1998 ± 272 and 2178 ± 278 m (1σ), (Figs. 2c, S3; Table S2). Both the estimation of surface uplift are consistent, suggesting the basin has experienced at least ~2000 m of net uplift. The paleo-relief of the basin estimated from the reconstructed paleo-channel suggests that the basin relief in the Dibang system has increased by 94 ± 13% since the onset of the current transient regime, which is responsible for the growth and upstream migration of knickpoints in the Dibang catchment. The relief increment in each tributary is consistent, which suggests the surface uplift as a regional phenomenon despite varying topography (Table S2) and variable drainage divide across the basin (Fig. 3a). The modelled paleo-channel profile along the elevated low relief sub-catchment (Db 7.2) matches remarkably well with the persevered relict reach in hanging tributaries (Fig. S1c) possibly suggesting they represent the relict surface prior to the new transient regime of Dibang basin. The paleo-base level (present elevation – net surface uplift) of the Db7.2 at wind-gap/coincident drainage divide^12^ is estimated to be ~1600 m (Figs. 2, S3) prior to the onset of the transient regime in Dibang basin.

### Validation of slope-break knickpoints

The 3k knickpoints retreat rate modelling in 37 channels of Dibang basin is carried out to test their co-genetic and mobile nature using celerity model^40,42,43^. The basic premise of celerity modelling is that knickpoint retreat rate (*dx/dt*) relates to contributing area (*A*) through non-dimensional exponent (*p*) and erodability coefficient (***C***)^42^

2 $$\frac{dx}{dt}=C{A}^{p}$$

A brute-force, two-dimensional grid search is carried out to find the best-fit ***C*** and ***p*** parameters (in the least square sense) between the observed and modelled knickpoint positions. The knickpoint celerity model is run for the 2–10 Ma time range, same as volume-for-time substitution method with a sampling interval of 0.25 Ma. The model shows <2% difference in the sum of least squares residual between observed and modelled knickpoint locations at any given travel time (Fig. S1b). The best-fitting p-parameter values for the 8 knickpoints in the elevated low relief landscape (Db-7.2 sub-catchment; Figs. 1b, S1b) range between 0.51 and 0.54 (Fig. S3a), whereas for the rest of the Dibang basin it varies from 0.42 to 0.46 (Fig. S3b). The erosional coefficient (*C*), varies over nearly an order of magnitude to accommodate the modelled knickpoint travel time. The observed and modelled knickpoints coincide remarkably well (Figs. 3b, S1b) validating the initial assumptions of their co-genetic origin and mobile nature.

### Constrain on perturbation timing

In absence of erosion/incision rate estimate for Dibang basin and the presence of comparable 3k knickpoints in the Yarlung-Siang system (Fig. 1b), the knickpoint retreat and travel time is being estimated based on paleo-base level (knickpoint) modelling with the long-term background exhumation rate in the Yarlung-Nyang confluence zone, upstream of Tsangpo gorge^5,30,46^. We applied volume-for-time substitution approach^40,41^ to the Himalayan wedge system (Fig. 2a) where the time is taken to erode the rock volume downstream of 3k knickpoint is same as the time taken to migrate the knickpoint from the mountain front since the onset of the transient state (Fig. 3a). The knickpoint travel time (*T*_*k*_) estimate depends on the eroded volume (*V*), underneath area (*A*_*c*_) and denudation rate (*E*_*r*_) as under

3 $${T}_{k}=(\frac{V}{{A}_{c}}){{E}_{r}}^{-1}$$

**Figure 4:**
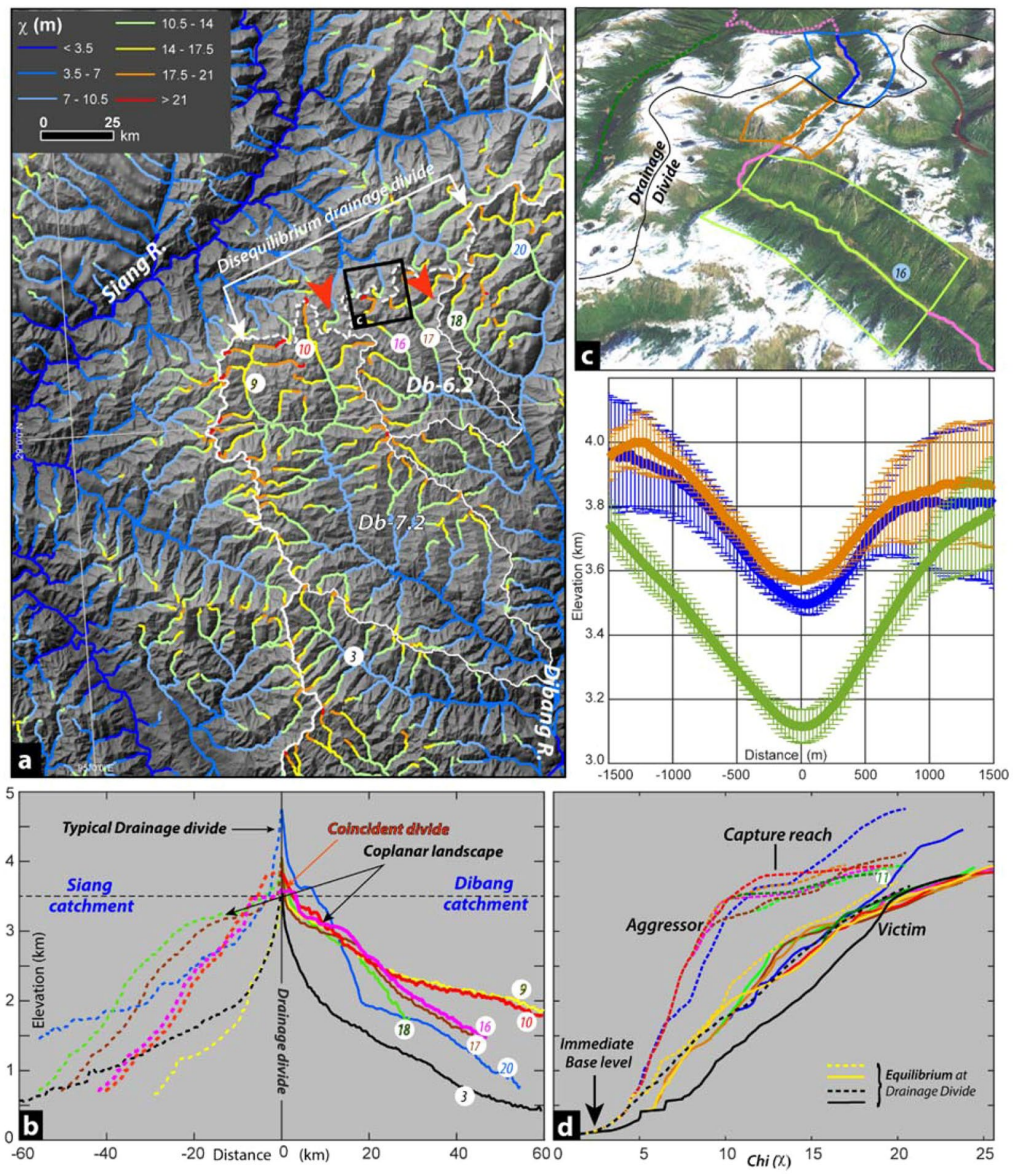
(**a**) The Chi (**χ**)-map of Siang-Dibang drainage divide region show the divide is in disequilibrium and migrating towards Dibang catchment. (Also see SM6). The tributaries across wind gap at the divide used for further analysis. (**b**) River profiles of tributary channels across the fixed drainage divide. The dotted and solid profile are tributaries of Siang and Dibang rivers, respectively. The profiles of Db. 7.2 and Db. 6.2 (red and pink, respectively) show unique flat relict landscape at ~3600 m elevations and co-planar landscape on either side of the divide. (**c**) The stacked swath profile with 1σ variation of flat relict landscape show broad misfit channel floor across the divide. (**d**) **χ**-elevation profiles across drainage divide clearly suggests that the tributaries of Siang are aggressor and encroaching the drainage area of victim Dibang catchment. Note the equilibrium behavior of yellow & black channels suggesting the extent of water divide.

We derived knickpoint travel time for a range of background erosion rates ranging from 0.1 to 0.4 km/Ma, with an interval of 0.05 km/Ma (Table S3). The knickpoint initiation time is projected to be 1.90–8.96 Ma with 3.3–4.2 Ma for the Dibang basin (Fig. 3c) for comparable denudation rate/knickpoint retreat rates of 0.25–0.20 km/Ma along the Yarlung river^30^. This could suggest the minimum initiation time of the transient state in the Dibang basin. The mean knickpoint velocity estimate for both the groups are within the range of measured knickpoint propagation rates for the bedrock rivers^47^ (Fig. 4c). By considering a similar range of knickpoint retreat rate of 20–40 km/Ma along the Yarlung^30^ and Dibang basin, the minimum initiation timing for the knickpoint growth and associated transient regime is projected to be 3–6 Ma (Fig. 3c). The knickpoints retreat rate in the elevated low relief landscape (Db-7.2) is ~15% faster from the rest of the basin (Fig. 3c).

### Drainage divide anomalies

In the ongoing transient state of the uniformly evolving Dibang basin, the western Siang-Dibang drainage divide lies at ~4000 m mean elevation, which is ~800 m lower than the eastern Dibang-Lohit segment with a wind gap zone (Fig. 3a). This segment in the Siang-Dibang drainage divide coincides with the crest of elevated low relief sub-catchment Db7.2 at ~3800–4000 m elevation (Fig. 4a,b). To understand the Siang-Dibang divide behaviour and possible place of drainage linkage through the wind gap segment with its surroundings, the tributary channel profiles are analyzed across fixed drainage divide (Fig. 4a,b). The extent of the wind gap region is marked by the channel nos. 3 and 20, which show a typical drainage divide profile with high gradient peak (Fig. 4b). The generalized stacked swath profiles with 2σ elevation^48^ across drainage divide of three segments of channel nos. 16 (Fig. 4c) and 10 mark a flat divide with misfit channel segment at elevation <3600 m. A few small glacial valleys with identifiable tarns at the ridge crest without much depositional landscape are observed in the region. They appear to be the Late Pleistocene-Holocene glacial landscape; however the equilibrium line altitude during last glacial maxima in the eastern Tibet and Himalaya is mapped & modelled to be at >4200 m amsl^49^. Therefore, the misfit channels observed at the drainage divides are part of the gently sloping relict reach above knickpoints and were occupied during the Holocene glaciation, rather than the products of glaciation. The celerity modelling clearly demonstrates that the cogenetic knickpoints have migrated from the mountain front at a lower elevation however most part of the observed elevated low relief relict landscape is situated <3000 m (Fig. S1c) and may probably represent remnant paleochannel. Further, the glaciation would have inhibited the erosion in the relict reach segment^49^ thereby making the incision more pronounced in active fluvial segment with enhanced stream power and producing higher *k*_*sn*_ (Fig. S1c).

The coplanar landscape across flat wind gap with misfit paleochannel, representing coincident drainage divide has contrasting landscape in the adjacent catchments viz., presence of elevated low relief landscape (Db 7.2 and Db-6.2; Figs. 1d, S1a,c) towards Dibang and higher relief towards the Siang river (Fig. 4b,c). This point towards an active disequilibrium across the drainage divide, which is invariably attributed to the drainage area exchange^11^. To examine the current state of drainage-divide stability and potential zones of drainage area exchange across Dibang and its neighboring basins, we carried out χ-analysis by transforming the χ-coordinate with reference to the horizontal coordinate (x) of river profile^11,13,50,51^. By integrating Eq. 1 after separating the variables and treating U and K as constant in space and time, the steady-state solution is achieved,

4a $$z(x)={z}_{b}+{k}_{s}{(\frac{1}{{A}_{0}^{m}})}^{\frac{1}{n}}\chi $$

4b $$\chi ={\int }_{{x}_{b}}^{x}{(\frac{{A}_{0}}{A(x)})}^{\frac{m}{n}}dx$$

where z_b_ is elevation at the drainage network’s base level at x = x_b_ and A_0_ is an arbitrary reference area. The χ-parameter values for the region are calculated for θ_ref_ = 0.45 and A_o_ = 1 m^2^ so that the slope in the χ plot as for the k_sn_ (Fig. S2). The high χ contrast is noted across the proposed wind gap of the Siang-Dibang divide (Fig. 4a) indicating unstable drainage divide where the high χ value of Dibang tributaries (10, 16, 17, 18 and 20 channels) suggest that it is losing drainage area to the corresponding tributaries with lower χ−value of Siang basin (Fig. 4d). This is a typical case of area loss feedback (ALF)^8,11,12,25,52^, where the channels of the expanding basin (aggressor = Siang) encroach the area of the receding basin (victim = Dibang). The head of Db 7.2 sub-catchment with elevated low relief landscape (Fig. S1), coincides with the wind-gap in Siang-Dibang divide (Fig. 4), forms the locus of channel diversion/ abandonment, thereby leaving behind beheaded Dibang basin.

## Discussion

**Figure 5:**
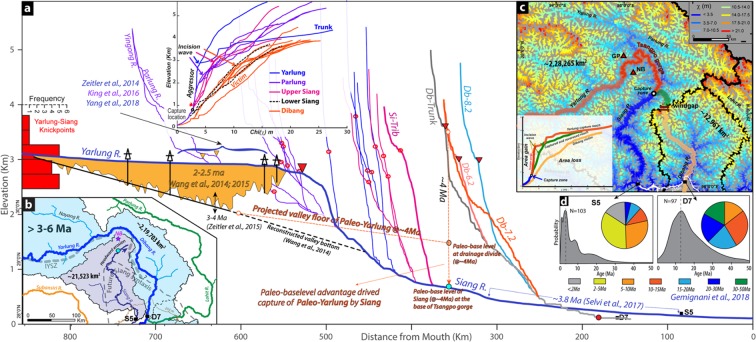
(**a**) Regional correlation of channel profiles of the Dibang catchment with the Yarlung-Siang system. The prominent 3k knickpoints are observed in Dibang and Yarlung drainage system. Note the χ profile of tributary channel in syntaxial zone clearly show the lower Siang and Dibang are in equilibrium, whereas the upper Siang, Yarlung and Parlung are clearly the aggressor channels. The ~4 Ma paleo-base levels of 3k knickpoint of Dibang river matches with the base level of paleo-Yarlung graded channel profile. Note the paleo-base level advantage of Siang river might have facilitated the capture of Yarlung. (**b**) Probable paleo-course of the Yarlung river during early Mio-Pliocene. (**c**) The χ map and profile of the Yarlung-Siang and Dibang drainage system clearly show drainage reversal of Upper Siang/Paleo-Yarlung at the coincident drainage divide and the Yarlung remain very active in the Tsangpo gorge driving the gradation processes in the region. (**d**) Note the provenance contribution by older grains at Dibang mountain front in comparison to the Siang.

Considering surface uplift-incision, landform growth, including drainage pattern are regional phenomena, we attempted to correlate the landform anomaly in Dibang basin with the adjoining region. The *in-situ* growth of the Dibang basin, which has been experiencing new transient surface uplift-incision regime since 3/3.5–6/7.5 Ma (Fig. 3c) producing co-genetic headward migrating 3k knickpoints from the mountain front (Figs. 3b, S1b). This clearly suggests that the relict reach (Figs. 1, S1a) represents a landform component formed prior to the onset of new transient regime. The comparable elevated low relief sub-catchment (Db 7.2 and 6.2) experiencing active incision, marked by low *k*_*sn*_ and hanging valleys (Fig. S1c) in the new transient regime of Dibang basin, represents a modified relict landscape^8,12,25^. This elevated low relief relict landscape zone is also the locus of ALF with drainage divide migration towards Dibang basin along the relict paleo-channel (Figs. 4, S1). The estimated timing of the transient state initiation in Dibang basin closely coincides with the initiation of rapid steepening of Tsangpo gorge with knickpoint at ~2820 m elevation started at ~ 4 Ma^5,6,53,54^. To understand the possible paleo-linkage of the >3–6 Ma old elevated low relief landscape (paleochannel) in the Dibang basin with the adjacent Yarlung-Siang river across coincident divide, the mobile nature and retreat rate range of coplanar 3k knickpoints along the Yarlung river and its tributaries in the Namcha Barwa region are analyzed (Figs. 5a, S5). The ***C*** and ***p*** parameters of the celerity model are consistent for four selected locations downstream of wind-gap zone for each model run, for the time range of 2–10 Ma with 0.5 Ma interval, suggesting the co-genetic and mobile nature of the knickpoints (Figs. 1b, 5a, S5). The modelled knickpoint initiation points with retreat rate 20–40 km/Ma for 3–6 Ma age bracket (similar to the knickpoint migration in the Dibang basin abandonment/isolation) matches for location-4 at the base of Tsangpo gorge (Figs. 5, S5). The location – 4 coincides with the aggressor channels in the upper Siang basin across the coincident drainage divide (Fig. 4) and marks an optimal convergence zone for the possible paleo-linkage of Yarlung river with the elevated low relief paleo-channel in the Dibang basin (Fig. S5) prior to isolation/abandonment. We carried out chi (χ) analysis of main tributary channels of Yarlung-Siang and correlated them with those of the Dibang basin across the wind gap region (Fig. 5a). The tributaries of the lower Siang (downstream of location – 4) are in gradational equilibrium similar to the Dibang basin (Fig. 5a inset), whereas the tributaries of upper Siang, Parlung and Yarlung with co-genetic ~3k knickpoints (Fig. S5; upstream of location – 4) are clearly in disequilibrium with the Dibang and share an aggressor-victim relationship (Fig. 5a inset). The χ- profiles of the major channels are consistent with the river capture model^26^, where the Dibang river (victim) has anomalously high χ -value at lower elevation, consistent with the area loss, whereas the upstream channels of Yarlung-Siang (aggressor) have low χ -value at higher elevation, consistent with the area gain (Fig. 5c). The higher χ value of victim Dibang river and the low χ value of aggressor upper Siang and Yarlung rivers is clearly in disequilibrium responding to the very high incision along the Tsangpo gorge (Fig. 5c inset), thereby driving the regional gradation process.

Prior to the steepening of Tsangpo gorge during Mio-Pliocene^6,7^, the Yarlung river with graded profile was flowing at a paleo-base level of ~2000 m upstream of the Tsangpo gorge^6^ (Fig. 5a). The paleo-base level at the Dibang-Siang coincident drainage divide/wind-gap is estimated to be ~1600 m (Figs. 3, 5, S3) prior to the onset of transient regime at 3–6 Ma ago (Fig. 3). If we extend the graded profile of the Yarlung paleo-base level, from upstream of Tsangpo gorge^6^, to the paleo-base level at the coincident drainage divide, lying >150 km downstream (Fig. 5a), it would form a steady-state Yarlung-Dibang graded paleochannel with uniform paleo-slope (Figs. 5a, S6a). Coincidently, this drainage divide at ~1600m paleo-base level during 3–6 Ma lies adjacent to the headward eroding Siang river at a lower base level, which was eroding the hinge zone of growing Siang syntaxis during <3.8 Ma^34^ (Figs. 1, 5a,b, S6a).

We analyzed the spatial coincidence of paleo-base levels of Yarlung, Dibang and Siang rivers during Mio-Pliocene to understand the role of drainage piracy in producing present geological scenario as it is variously observed that the river capture act as a driver of transient landscape evolution^5,22,39^. The headward eroding Siang river would have captured the paleo-Yarlung-Dibang river owing to a lower base level (>700 m) advantage thereby setting in a new transient landscape evolution process in the Namcha Barwa region (Fig. S6b). The capture of paleo-Yarlung-Dibang river by the Siang river would cause ~17 times loss in drainage area and drainage discharge to Dibang basin leading to a decrease in erosion rate by 4–17 times considering 0.5 < m < 1; ∆E = ∆A^m 8^ (Fig. 5b,c). This would result in an increase in net surface uplift (as E < U) along the course of beheaded channel (sub-catchments Db-7.2, 6.2) to evolve as elevated low relief landscape (Fig. 5,S6b)^9,10^. In post-capture scenario, the abandoned Dibang basin would initially experience net surface uplift (E < U) followed by evolution in new transition state trying to attain steady-state (E = U) with a wave of transient signal originating from the mountain front, which evolved as the 3k knickpoint and associated landscape since 3–6 Ma ago (Figs. 5c, S6c,d). The high stream power in the mountain front also suggests the tectonics in the front is driving the growth and evolution of transient signal in the Dibang basin (Fig. S1c). The evidence for this proposed drainage piracy of the paleo-Yarlung-Dibang river by the Siang river is also reflected in the detrital age distributions in the foreland molassic deposits at the mountain front of the Siang and Dibang rivers^35^. The sediments at the Dibang mountain front (D7) is constituted of only >5 Ma old detrital zircon, in contrast the Siang (S5) is dominated (>50%) by <5 Ma grains population (Fig. 5c). This clearly suggests the Dibang was connected to the large upstream catchment area, which got disrupted before ~5 Ma. This concurs with our interpretation of the Dibang being part of Mio-Pliocene Yarlung paleochannel, which was captured by river piracy along the Siang river. Further, the dominance of <5 Ma detrital zircon^35^ and the zircons in Siwalik sediments along Siang river are derived from the Tethyan or Greater Himalaya source at the expense of Trans-Himalayan grains between 3.6–6.6 Ma^31^. This clearly negates the Siang river connectivity from the Trans-Himalayan region prior to the 3.6–6.6 Ma period^31^ and complement the present interpretation that the paleo-Yarlung was flowing through Dibang river as shown in the present study (Figs. 5, S6).

The late Miocene-Pliocene capture of the paleo-Yarlung-Dibang by the Siang river resulted in gain in drainage area and discharge with sharp drop of >700 m of base level. This would have accelerated the steepening upstream of capture zone leading to the formation of the Tsangpo gorge through focused exhumation at the rate of >5 mm/yr^46^ causing crustal thinning and tectonic aneurysm in the NB syntaxial zone during ~4 Ma^4,5^ (Fig. S6c,d). The sudden exhumation flux due to river capture would have led to the channel impoundment and sediment accumulation upstream of the gorge^6,54,55^ (Fig. 5a). The capture of paleo-Yarlung river by the Siang river and abandonment of Dibang segment has further implication on paleo-drainage pattern and sediment supply beyond Himalayan front. Prior to the river capture, we speculate that the paleo-Yarlung-Dibang river would have drained to the Surma basin through trough between the Mikir hills and Indo-Burmese ranges^56^. Alternatively, the paleo-Yarlung river may have drained through the Irrawadi (Fig. S9) if we consider the presence of very wide abandoned channel at <1000 m elevation in the Indo-Burmese range. The later assertion requires further landscape modelling and detrital provenance study.

## Conclusions

It is clear from the above discussion that the conspicuously symmetrical Dibang basin is undergoing transient evolution since 3–6 Ma. The unique elevated low relief relict landscape in the northwestern part of the basin represents the abandoned channel of Paleo-Yarlung, which was captured by Siang river during Mio-Pliocene period owing to lower (by >700 m) base level advantage. This channel piracy caused ~4–17 times drainage area and discharge gain to Siang at the capture point leading to rapid incision and development of Tsangpo gorge that caused crustal thinning and tectonic aneurysm^4,5^ producing very high exhumation in Namcha Barwa region. The recent studies on detrital sediments in the foreland deposits^34^ also support the large-scale drainage reorganization and capture of Paleo-Yarlung by Siang during Mio-Pliocene as demonstrated in the present study.

## Methods

### Drainage network extraction, terrain and river profile analysis

The drainage network was extracted from the 30 m ASTER and 90 m SRTM digital elevation model (DEM) (10.5067/ASTER/ASTGTM.003) in ESRI ArcGIS platform. To remove the artifacts and noise embedded in the elevation data, we have used 1,000 m smoothing window and the 20 m vertical interval to sample the channel networks. A regional relief map (Fig. 1b) was generated by passing 500 m circular radius focal range window over the 30 m horizontal resolution DEM and regional slope map (Fig. SM1a) was created by passing 500 m radius mean filter over the slope model extracted from the DEM. The DEM and the extracted flow accumulation data is used in MATLAB with the modified stream profiler tools^22^ for further analysis (Figs. 1, S1; http://geomorphtools.geology.isu.edu/index.htm). Only the fluvial-dominated channel segments were used for the knickpoint analysis and we used ~1 × 10^6^ m^2^ accumulation area as cutoff value from hillslope dominated channels to fluvial dominated channels^26,57,58^.

The persistent break in the linear pattern in the log scaling relationship between drainage area and slope is identified as slope-break knickpoints^26,57^ and the above property is used to delineate slope-break knickpoint (Fig. 1). To investigate the variation in tectonic uplift and/or erosion, the Steepness index (Eq. 1) and Normalized steepness index (*k*_*sn*_ were computed at reference concavity (*θ*_*ref*_), which allows a fair comparison across the basin despite having greatly varying basin area (Wobus *et al*., 2006). To determine *θ*_*ref*_, we followed the method of least scattered chi (*χ*)-profile^59^ where we have considered more than 300 channels of Dibang basin and calculated the Chi (*χ)* profile for a range of *θ* (0.05 to 0.7). We sub divided the *χ*-space into 100 bins and estimated the corresponding mean of the distribution over standard deviation of elevation. The degree of scatterings of *χ*-profile is minimum at *θ* = 0.4 (Fig. SM2). However, in order to determine the regional *k*_*sn*_ and *χ*-map for the entire Yarlung-Siang and Dibang basins, we have used *θ* = 0.45 for the present study as it encompasses the catchment beyond Dibang and the results could be comparable to other previous studies^30,46,60^.

We computed the *k*_*sn*_ for drainage networks in eastern Himalaya using the empirical relation (*k* = *SA*^θref^. The local slope were calculated along the profile at 20 m contour interval and the K_sn_ averaged at 0.5 km interval (Figs. 3, SM3). The radial retreat pattern and the similar base level height of 36 knickpoints supports the mobile slope-break knickpoint system in the Dibang catchment^42,43^. These knickpoints are moving upstream like a kinetic wave and the highest set of knickpoints demarcates the boundary between relict reach and the lower actively readjusting basin^45^. The presence of mobile knickpoint suggest that the Dibang basin is in transient state^61^.

### Paleo-channel Reconstructions

The paleo-base level of the longitudinal river profiles were reconstructed based on the assumption that the modern surface hydrologic network is not varied significantly since the onset of the transient state. The reconstruction of paleo-base level elevation were calculated using the Eq. 1 from the knickpoint to the mountain front^17,40,45,62,63^. The power law scaling parameters *k*_*sn*_ and mean *θ*_*ref*,_ were estimated from the relict segment of the upstream to the knickpoint. The theoretical elevation of paleo-base level estimated at each pixel along the flow accumulation path from the top of the slope-break knickpoint to the mountain front (MF) of a drainage basin. The upper and lower boundary of the reconstructed paleo-base level were computed for each longitudinal profile based on the 2σ errors in the estimation of normalized *k*_*sn*_. The paleo-base-level error was assigned as the dotted line figure (Figs. 3, SM3; Table SM2). The net surface uplift (Δ*z* = *U* *−* *E*) and paleo-relief were estimated for each channel by subtracting the modern base-level elevation and highest elevation from the paleo-elevation at the mountain front.

### Knickpoint retreat rate by Celerity Model

The distribution of the 3k knickpoints were modeled using the celerity model Eq. (3), which determine two unknown parameters *‘C’*-detachment-limited erosion coefficient and *‘p’*- drainage area exponent through brute-force search approach^40,42,43^. The *C* parameters was set to vary from 10^−10^ to 10^−4^ over 500 increments, while *p* values were set to vary from 0.2 to 1.2 over 40 evenly-spaced increments (Fig. SM6a,b). The resulting summation of least square residuals for each group of knickpoints for modelled travel time was used to determine the best fit *C* and *p* combination, which are used to estimate the mean horizontal knickpoint retreat velocity.

## Supplementary information

Supplementary figures
