# Supplementary material for: Mio-Pliocene piracy, relict landscape and drainage reorganization in the Namcha Barwa syntaxis zone of eastern Himalaya
Source: Sci Rep. 2019 Nov 26;9:17585. doi: 10.1038/s41598-019-54052-x (PMC6879501; doi:10.1038/s41598-019-54052-x)
Supplement: Supplementary file 1 — Supplementary figures [file 41598_2019_54052_MOESM1_ESM.pdf]

Mio-Pliocene piracy, relict landscape and drainage reorganization in the Namcha Barwa syntaxis zone of eastern Himalaya

Nilesh Kumar Jaiswara, Prabha Pandey, Anand K Pandey §

Academy of Scientific Innovation and Research, CSIR-National Geophysical Research Institute, Uppal Road, Hyderabad - 500007, India

N.K. Jaiswara : nilesh_riit@yahoo.co.in

P.Pandey : ppandey@ngri.res.in

§ Corresponding author: akpandey@ngri.res.in

EXTENDED DATA

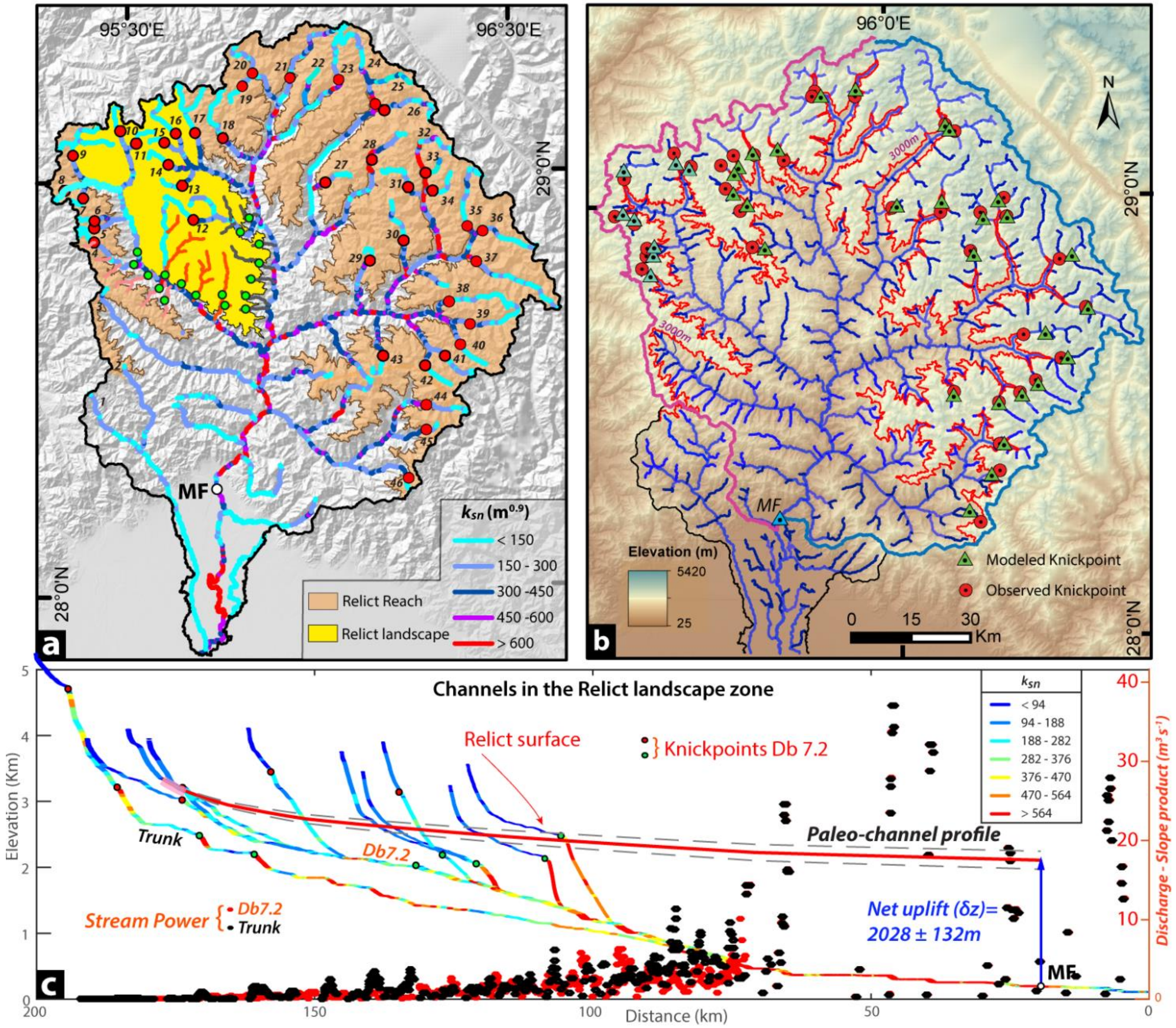

Fig. S1 (a) The k_{sn} map overlaid with 3K knickpoints (red dots) of Dibang basin are analysed to characterize the relict reach and active segment of channels profiles in the transient basin. The elevated low relief relict landscape region is marked in yellow. Note the numbering scheme of 46 tributary channels. (**b**). Spatial distribution of observed and modeled 3k knickpoints by celerity modelling in Dibang basin for 3.75 Ma. (**c**) The channel profiles of the Trunk and elevated low relief relict landscape region (Db 7.2) overlaid with the ksn, knickpoints, paleo-channel reconstruction and stream powers along the respective zone.

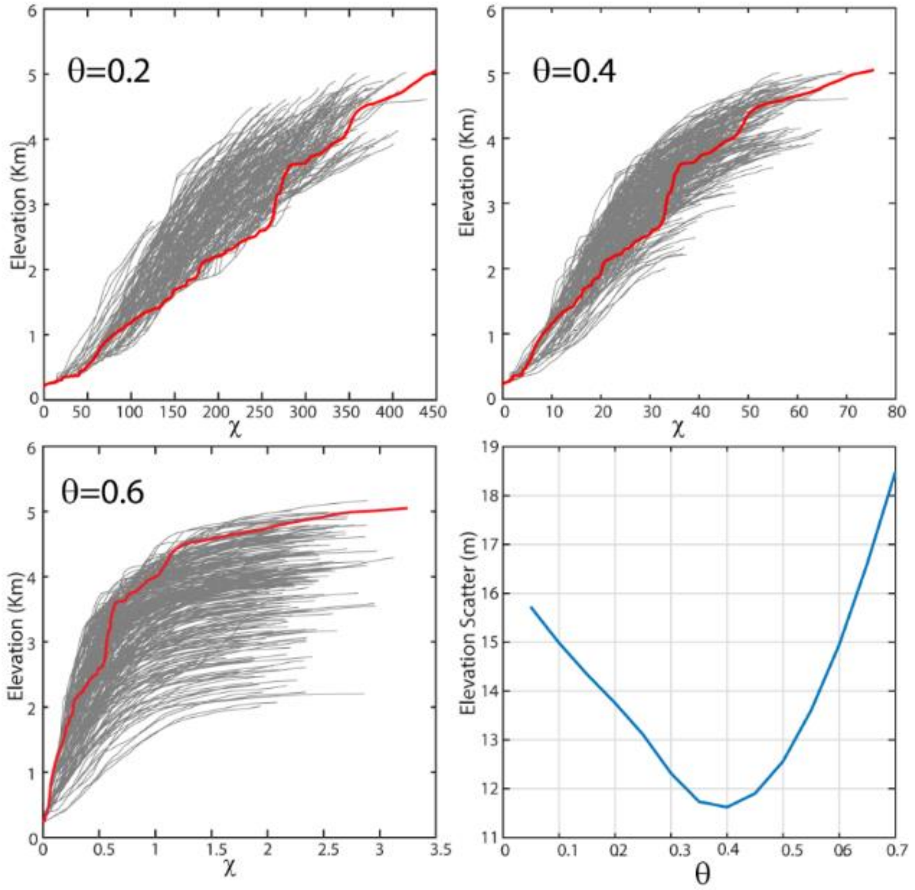

Fig. S2: Chi (χ) - elevation plots of tributaries of Dibang river based on a range of θ values = 0.2, 0.4, 0.6. The trunk channel is shown in red. Degree of elevation scatter in χ–profile as a function of θ shows minima at θ=0.4. The χ–values are calculated with A^0 = 1 m2.

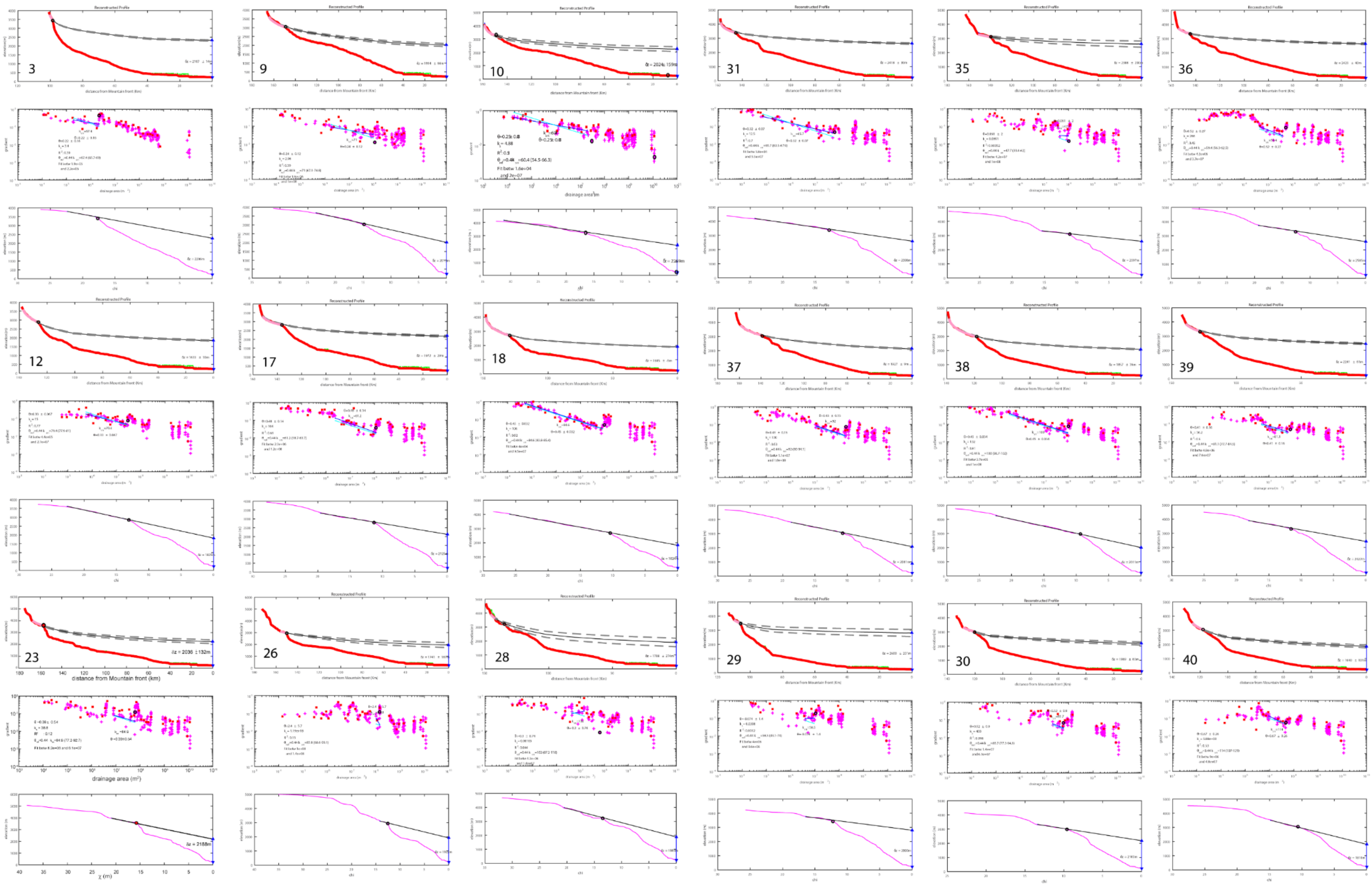

3
9
10
31
35
36
12
17
18
37
38
39
23
26
28
29
30
40
distance from Mountain front (km)
drainage area (m²)
χ (m)

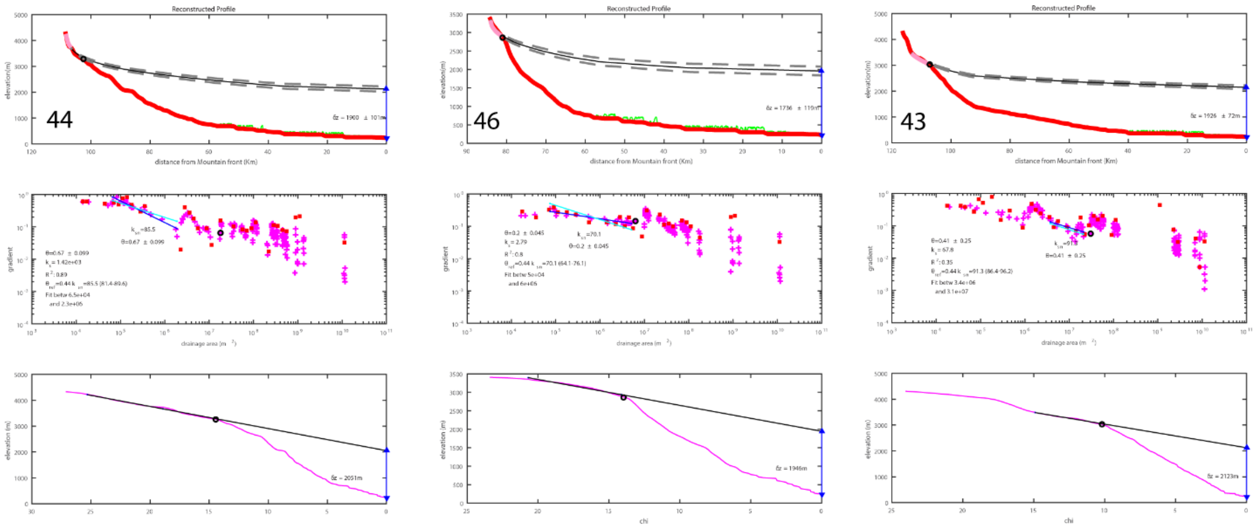

S3 Reconstructed paleo-base level of 21 tributaries with only well persevered relict reaches for reconstructing the spatial variations in the net surface uplift in Dibang basin.

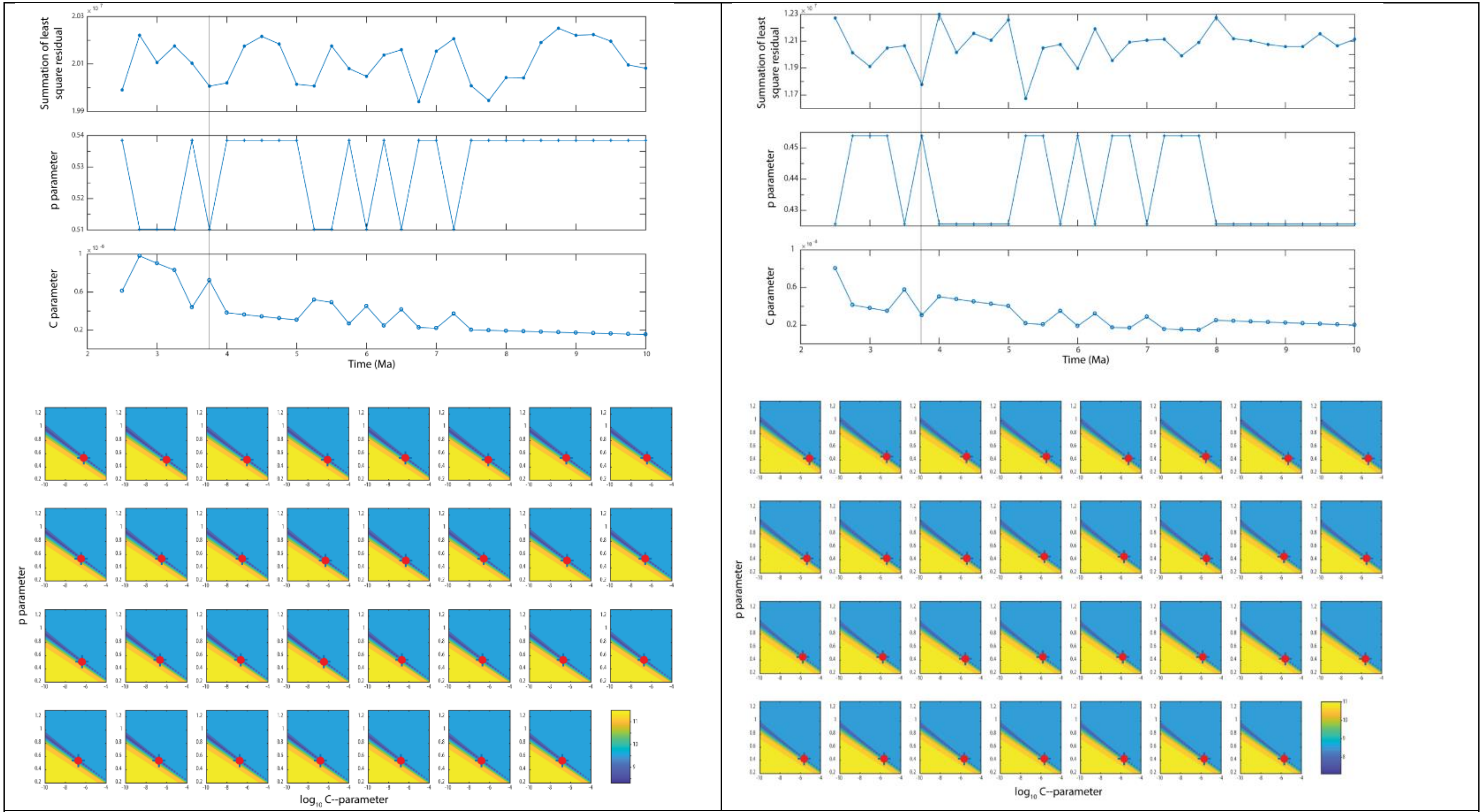

Fig. S4: C and p parameters in celerity modeling for tributary channels with 3k knickpoints are estimated from brute-force two-parameter search method (Crosby & Whipple, 2006; Berlin and Anderson, 2007; Gallen et al. 2013). The celerity models for two group of channels (a) 29 channels belonging in rest of the Dibang basin, (b) 8 channels from elevated low relief Db-7.2 sub-catchment for the time range of 2.5-10 Ma with 0.25 Ma interval. The smallest residual value for each model run is marked by red dot and the misfits are minimized within a narrow range.

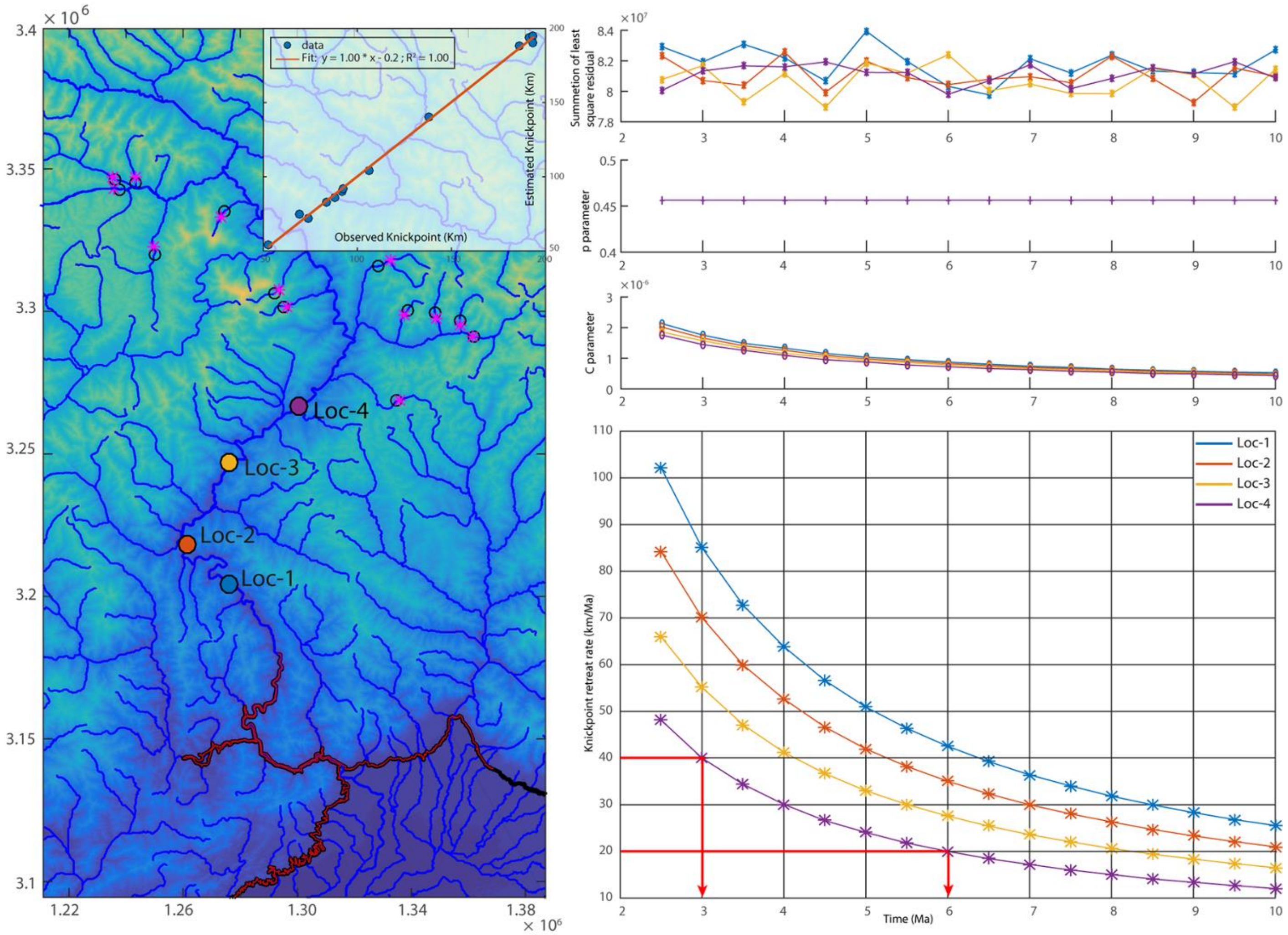

Fig. S5. The mobile nature of thirteen 3k knickpoints in the Yarlung-Siang river have tested through the celerity model. The C and p parameters are consistent for age range 2-10Ma (with interval of 0.5Ma) from four selected location at the downstream of NB gorge. The Correlation of observed and modeled knickpoint distances from Loc-4 for 4Ma age is shown in inset. We excluded the knickpoints from the Parlung river as it has joined the Yarlung river much later (<1 Ma [31,33]) and NB knickpoint as the retreat has hindered by steepening of gorge. The effect of the Parlung drainage area on celerity model was taken care by considering area for only last 1Ma.

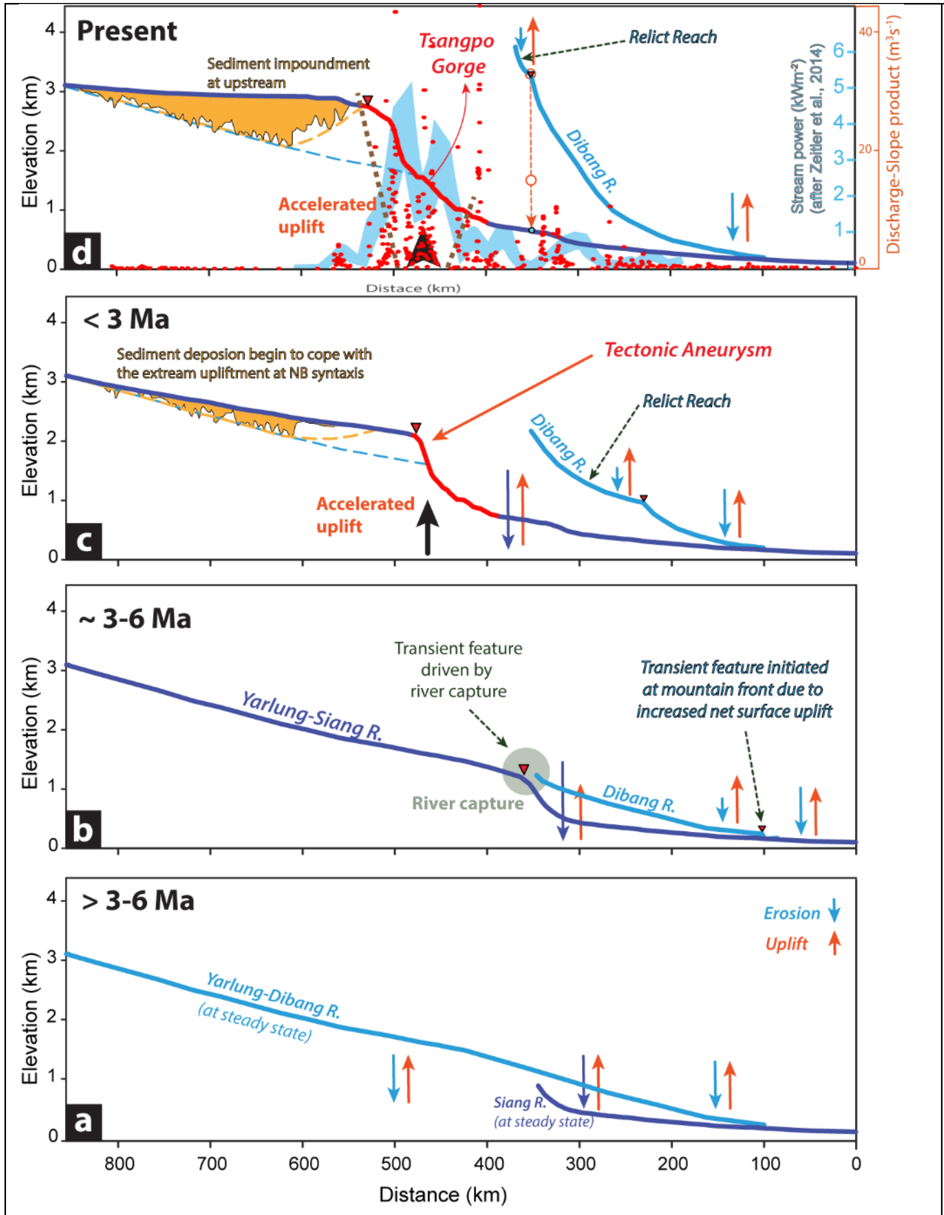

Fig. S6: Proposed model for drainage evolution and reorganization in the eastern Himalayan syntaxis zone. (a) The Dibang constitute the frontal segment of Paleo-Yarlung prior to ~4 Ma with graded profile. (b) The rapid incision and headward migration of Siang river at a lower base level due to Siang syntaxial growth during ~3.8 Ma led to the capture of paleo-Yarlung aided by positive feedback between rapid uplift and erosion at NB massif. The capture of Paleo-Yarlung decreased the erosional efficiency of perched abandoned segment, identified as Db7.2 and created a new transient state in the Dibang basin. This disequilibrium in uplift-erosion triggered the initiation of slope break knickpoint at the mountain front. (c) The headward knickpoint migration along the Dibang tributaries led to the growth of relict reach in intermediate stage. (d) Present day drainage system in eastern Himalayan syntaxis with knickpoint distribution, which is modeled to get ~2 Km of net uplift and minimum timing of channel abandonment.

Table S1: Characterization of relict reach and active segment of tributaries of Dibang basin. Note that the tributaries of Db-7.2 shows lower mean K_{sn} value as compare to rest of the tributaries.

	Upper reach				Lower reach							
**Channel No.**	**Concavity(θ)**	**Steepness index (k_s)**	**R^2**	**Normalized Steepness index ($k_s \pm 2\sigma$)**	**Concavity(θ)**	**Steepness index (k_s)**	**R^2**	**Normalized Steepness index ($k_s \pm 2\sigma$)**	**Knickpoint elevation (m)**	**Mountain front base level (m)**	**Horizontal retreat (km)**	**Vertical retreat (m)**
db-4	0.56 ± 0.33	618.04	0.471	118.07 ± 10.68	0.27 ± 0.05	9.28	0.426	272.41 ± 7.1	2516	255	103.52	2261
db-5	0.06 ± 0.21	0.25	0.028	85.3 ± 2.68	0.32 ± 0.05	23.5	0.536	249.53 ± 6.32	3178	255	121.58	2923
db-6	0.34 ± 0.22	16.49	0.366	88.95 ± 3.42	0.19 ± 0.13	1.6	0.085	296.34 ± 9.59	3317	255	121.04	3062
db-7	0.48 ± 0.18	170.95	0.697	111.91 ± 4.24	0.21 ± 0.11	2.1	0.105	237.75 ± 9.72	3306	255	136.92	3051
db-8	0.74 ± 0.33	9997.19	0.57	121.34 ± 6.33	0.24 ± 0.07	3.82	0.257	219.89 ± 7.73	3164	255	138.94	2909
db-9	0.33 ± 0.12	15.18	0.52	92.19 ± 3.43	0.21 ± 0.1	2.04	0.111	226.99 ± 8.12	3042	255	140.3	2787
db-10	0.56 ± 0.23	657.84	0.562	100.13 ± 7.15	0.23 ± 0.07	3.37	0.223	223.65 ± 7.01	3213	255	139.59	2958
db-11	0.34 ± 0.18	11.2	0.54	59.23 ± 3.77	0.16 ± 0.1	0.8	0.075	240.23 ± 8.75	3233	255	137.23	2978
**Mean of Db-7.2**				**97.14 ± 19.10**				**245.84± 24.72**				
db-12	0.4 ± 0.16	41.38	0.538	98.25 ± 1.88	0.32 ± 0.06	23.4	0.534	234.8 ± 6.58	2837	255	117.01	2582
db-13	1.15 ± 0.24	8029595.27	0.869	124.66 ± 10.82	0.37 ± 0.05	60.05	0.611	269.49 ± 3.34	3059	255	127.5	2804
db-14	0.6 ± 0.21	1024.47	0.692	83.82 ± 3.14	0.4 ± 0.06	114.15	0.598	276.52 ± 3.65	3079	255	129.2	2824
db-15	0.62 ± 0.2	1415.2	0.844	96.07 ± 4.12	0.35 ± 0.05	43.17	0.58	270.42 ± 3.05	3216	255	132.21	2961
db-16	0.74 ± 2.46	11645.41	0.066	58.7 ± 9.04	0.33 ± 0.06	31.1	0.53	269.21 ± 7.24	3012	255	132.18	2757
db-17	0.56 ± 0.15	601.16	0.723	72.07 ± 3.38	0.37 ± 0.07	73.14	0.469	277.37 ± 4.28	2838	255	128.13	2583
db-18	0.48 ± 0.09	189.95	0.752	101.05 ± 2.13	0.41 ± 0.07	162.86	0.535	290.78 ± 5.32	2697	255	120.74	2442
db-20	0.26 ± 0.13	7.19	0.321	179.39 ± 5.46	0.34 ± 0.1	33.44	0.261	248.86 ± 4.73	2737	255	141.64	2482
db-21	0.4 ± 0.12	102.15	0.45	217.1 ± 6.21	0.31 ± 0.11	19.69	0.209	232.03 ± 5.81	2496	255	136.92	2241
db-25	0.4 ± 1.22	50.15	0.067	78.3 ± 16.59	0.41 ± 0.1	158.97	0.323	226.82 ± 6.22	2654	255	140.64	2399
db-26	0.33 ± 0.27	21.57	0.165	158.18 ± 5.62	0.25 ± 0.12	4.86	0.14	217.62 ± 7.71	2982	255	141.81	2727
db-27	0.38 ± 0.23	54.98	0.326	164.94 ± 6.1	0.16 ± 0.19	0.63	0.035	299.28 ± 9.22	2972	255	112.15	2717
db-28	1.84 ± 1.34	3.15E+12	0.454	98.61 ± 24.95	0.33 ± 0.07	30.71	0.382	299.21 ± 9.02	3205	255	125.17	2950
db-30	0.85 ± 0.99	133164.38	0.212	102.02 ± 11.24	0.39 ± 0.18	117.38	0.195	360.36 ± 5.9	2995	255	111.33	2740
db-31	0.05 ± 0.48	0.12	0.003	85.87 ± 9.8	0.21 ± 0.17	2.22	0.066	350.32 ± 5.86	3343	255	135.89	3088
db-33	1.47 ± 0.31	2071123809	0.898	95.22 ± 15.07	0.25 ± 0.16	5	0.09	348.47 ± 5.99	3353	255	138.25	3098
db-34	0.56 ± 0.75	885.15	0.114	119.72 ± 12.26	0.25 ± 0.16	5	0.09	348.47 ± 5.99	3201	255	134.2	2946
db-35	0.56 ± 2.07	735.97	0.04	55.95 ± 17.49	0.38 ± 0.12	81.46	0.244	351.98 ± 3.87	3085	255	130.4	2830
db-36	0.35 ± 0.5	27.09	0.102	132.21 ± 7.5	0.4 ± 0.12	141.25	0.262	353.87 ± 3.76	2957	255	129.82	2702
db-37	0.4 ± 0.24	54.71	0.318	110.62 ± 4.06	0.27 ± 0.18	7.3	0.088	354.04 ± 5.54	2831	255	123.78	2576

db-38	0.39 ± 0.11	43.11	0.525	117.19 ± 3.43	0.44 ± 0.06	354.09	0.61	390.52 ± 4.66	2957	255	109.31	2702
db-39	0.2 ± 0.14	1.96	0.23	103.99 ± 8.24	0.0.39 ± 0.05	94.2	0.58	385.19 ± 6.27	2957	255	112.98	2702
db-40	0.53 ± 0.19	491.1	0.501	124.09 ± 2.56	0.42 ± 0.06	232.04	0.594	388.95 ± 4.16	3203	255	114.32	2948
db-41	0.58 ± 0.08	1318.26	0.833	164.45 ± 4.88	0.39 ± 0.05	130.01	0.617	383.94 ± 5.37	3080	255	109.57	2825
db-42	0.54 ± 0.27	467.09	0.447	89.54 ± 4.32	0.41 ± 0.05	196.39	0.648	387.17 ± 5.19	3205	255	109.74	2950
db-43	0.3 ± 0.32	10.47	0.175	103.72 ± 6.89	0.41 ± 0.04	178.56	0.742	370.46 ± 2.82	3029	255	98.15	2774
db-44	0.15 ± 0.12	1.1	0.132	163.62 ± 10.52	0.54 ± 0.16	2280.79	0.341	250.49 ± 12.82	2603	255	84.57	2348
db-45	0.26 ± 0.07	13.71	0.714	236.79 ± 9.6	0.47 ± 0.06	519.47	0.657	267.29 ± 8.36	2805	255	78.71	2550
db-46	0.23 ± 0.08	4.39	0.649	108.8 ± 5.22	0.47 ± 0.06	428.78	0.658	220.62 ± 6.78	2832	255	71.46	2577
**Mean of rest of the catchment**				**118.79±42.72**				**307.74±58.13**				

Table S2: Table 3.3: Reconstruction of paleo-base levels for tributaries of Dibang river which is used to estimate net surface uplift and paleo relief.

Channel no.	θ_{ref}	Normalized steepness index (k_{sn}) ($m^{0.88}$)	k_{sn}_std (±σ)	Area left (m^2)	Area right (m^2)	SA- Surface uplift (m)	Std (±σ)	Paleo relief (m)	Modern relief (m)	χ- Surface uplift (m)
3	-0.44	67.35	1.63	186098	2166371	2106.65	57.03	1596.54	3703.18	2286
9	-0.44	70.99	3.84	1574100	1.02E+08	1813.59	110.87	1909.60	3723.18	2013
10	-0.44	60.43	5.90	16445	19876500	2024.30	197.00	1843.88	3868.18	2269
12	-0.44	79.44	1.58	444477	2.71E+07	1633.48	60.07	1893.70	3527.18	1823
16	-0.44	49.19	4.11	22653900	68441400	2185.72	85.66	1616.46	3802.18	2370
17	-0.44	61.22	2.51	2300400	1.17E+08	1972.08	67.51	1765.10	3737.18	2125
18	-0.44	84.60	0.84	40222	94559772	1694.69	47.80	2281.50	3976.18	1824
23	-0.44	84.94	7.76	8156826	6.09E+07	2036.43	185.85	2801.75	4838.18	2188
26	-0.44	83.85	15.24	1.03E+08	1.42E+08	1741.12	239.92	3042.06	4783.18	1922
28	-0.44	102.50	15.29	4304556	14136156	1708.30	338.58	2766.88	4475.18	1883
29	-0.44	58.54	15.45	3.97E+06	9.57E+06	2600.29	268.06	1423.89	4024.18	2800
30	-0.44	85.70	8.37	13965875	60808593	1988.60	137.01	1954.58	3943.18	2183
31	-0.44	65.70	2.16	58174	54934277	2417.60	71.59	1756.58	4174.18	2598
35	-0.44	47.70	14.34	41906700	1.01E+08	2388.27	229.88	2101.91	4490.18	2597
36	-0.44	59.42	3.13	4205700	36835200	2422.90	77.87	2274.28	4697.18	2585
37	-0.44	92.02	2.04	10953900	1.79E+08	1926.76	66.44	2559.43	4486.18	2081
38	-0.44	100.29	1.63	373722	1.01E+08	1857.46	35.93	2673.72	4531.18	2011
39	-0.44	81.12	3.40	4908295	7.57E+07	2240.65	60.54	2042.54	4283.18	2427
40	-0.44	113.92	6.87	9001214	4.79E+07	1639.75	100.62	2681.43	4321.18	1810
43	-0.44	91.30	4.90	3382431	30678036	1926.10	72.06	2181.08	4107.18	2123
44	-0.44	85.52	4.10	65319	2321657	1899.72	101.00	2222.46	4122.18	2051
46	-0.44	70.14	5.99	50305	5982486	1736.35	118.86	1459.83	3196.18	1946

Table S3: Knickpoint travel time estimated by the volume-for-time substitution method.

Denudation rate (Km/Ma)	Maximum time (Ma)	Minimum time (Ma)	Mean time (Ma)	Horizontal retreat rate (Km/Ma)	Vertical retreat rate (Km/Ma)
0.10	8.96	7.61	8.29	14.72	0.33
0.15	5.98	5.08	5.53	22.08	0.50
**0.20**	**4.48**	**3.81**	**4.15**	**29.44**	**0.66**
**0.25**	**3.59**	**3.05**	**3.32**	**36.80**	**0.83**
0.30	2.99	2.54	2.77	44.15	1.00
0.35	2.56	2.18	2.37	51.51	1.16
0.40	2.24	1.90	2.07	58.87	1.33
